# Periocular fibrosarcoma with lipogranulomatous conjunctivitis in a cat

**DOI:** 10.1111/vop.13249

**Published:** 2024-06-11

**Authors:** T. M. Foster, G. M. Newbold, E. J. Miller, Y. J. Jeong, C. Premanandan, B. D. Husbands

**Affiliations:** ^1^ Department of Veterinary Clinical Sciences, College of Veterinary Medicine The Ohio State University Columbus Ohio USA; ^2^ Department of Veterinary Biosciences, College of Veterinary Medicine The Ohio State University Columbus Ohio USA

**Keywords:** conjunctivitis, feline, fibrosarcoma, lipogranulomatous, periocular, veterinary ophthalmology

## Abstract

A 9‐year‐old, female spayed domestic short‐haired cat was presented with a 4‐year history of bilateral lipogranulomatous conjunctivitis (LGC), which was confirmed via histopathology. Thirteen months following the initial biopsy, the cat was presented with a rapidly progressive mass lesion of the palpebral conjunctiva of the right eye. A surgical debulking, followed 1 month later by exenteration after marked regrowth of the mass confirmed fibrosarcoma. This case report is the first to describe a cat with chronic bilateral LGC that later developed a unilateral fibrosarcoma within the eyelid tissue of the right eye. Fibrosarcoma should be considered a differential in any cat with chronic LGC that develops a rapidly progressive mass in the eyelid.

## 1 INTRODUCTION

Lipogranulomatous conjunctivitis (LGC) is most commonly reported in cats and has a poorly defined etiology. This form of conjunctivitis is distinctively characterized by smooth white or pale‐yellow subconjunctival nodules or larger lobules in the palpebral conjunctiva of cats, with associated inflammation.^1^, ^2^, ^3^, ^4^ Lesions most often occur in the upper eyelid adjacent to the eyelid margin and may be unilateral or bilateral.^2^ Previous reports have suggested that these lesions are more common in older (mean age 11 years), white or light‐colored cats. While the hypothesis is that these lesions may be associated with solar damage (actinic radiation), the exact etiopathogenesis is unknown.^2^ One hypothesis is that trauma or inflammation may lead to damaged meibomian glands, causing leakage of lipid into the conjunctival lamina propria and resulting in further inflammation. This condition has been described as a form of chalazion but may be somewhat diffuse rather than focal within the eyelids. While medical management with topical or systemic anti‐inflammatory drugs may yield limited benefit in some patients, surgical excision can be curative.^1^, ^2^

Lipogranulomatous conjunctivitis has been described along with concurrent ocular conditions, such as entropion or corneal ulceration. These ancillary findings are suspected to be secondary to irritation from the conjunctival nodules. However, neoplastic changes have also been described in conjunction with LGC.^1^, ^2^ The most often described concomitant neoplasm is squamous cell carcinoma; however, an undifferentiated orbital sarcoma and a mucinous carcinoma have also been described in association with LGC.^1^ The case report herein describes a cat with a chronic history of bilateral LGC that eventually was diagnosed with unilateral palpebral fibrosarcoma.

## 2 CASE HISTORY

A 9‐year‐old, female spayed domestic short‐haired, darkly pigmented, gray‐colored cat was presented to the Ohio State University College of Veterinary Medicine (OSUCVM) Spectrum of Care Clinic for a dental procedure. At that time, the cat had an owner‐reported 4‐year history of chronic, bilateral conjunctivitis, and eyelid thickening. Previous treatments included intermittent topical ophthalmic dexamethasone and tobramycin solution in both eyes (OU). Physical exam findings outlined proliferative conjunctival tissue OU, with the right eye (OD) more affected than the left eye (OS) and mild epiphora OU. A conjunctival biopsy was performed OD at the time of the dental procedure and was submitted to the OSUCVM anatomic pathology service.

**FIGURE 1:**
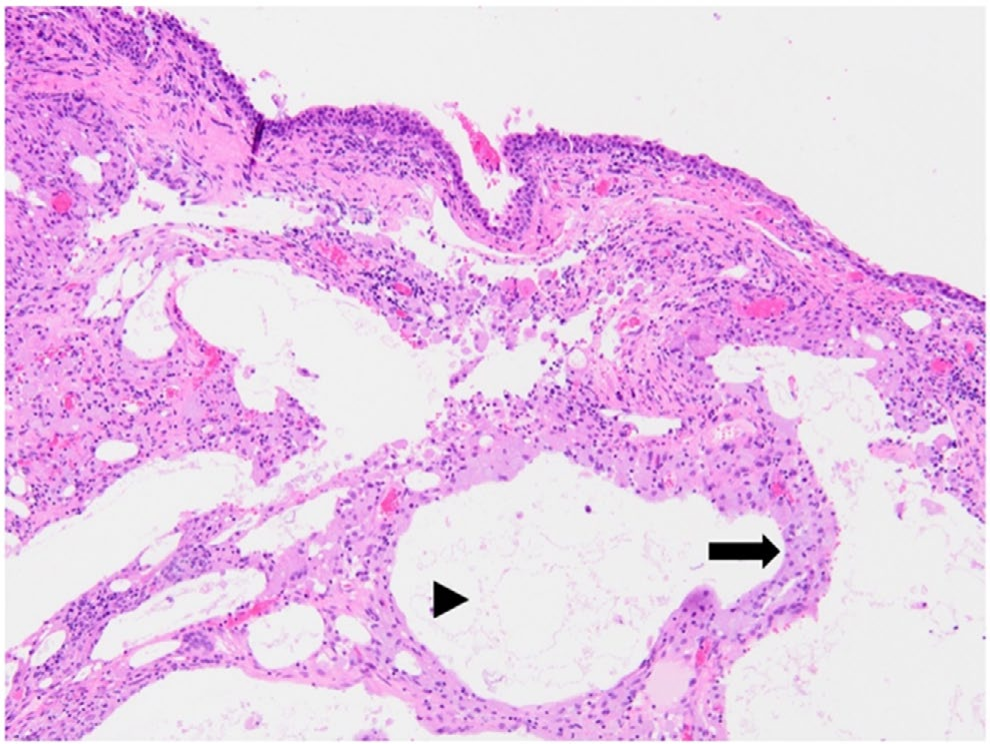
Initial histopathological image. Lipogranulomatous conjunctivitis is characterized by multifocal clear spaces (lipid) expanding the conjunctival muscularis (arrowhead) surrounded by aggregates of plump macrophages (arrow) 10X. Sample was taken from palpebral conjunctiva OD at initial presentation for dental procedure.

Histopathology demonstrated large clear spaces containing amphophilic glassy material and occasional cholesterol clefts within the conjunctival substantia propria. In these areas, plump macrophages containing small lipid vacuoles and multinucleated cells with up to 21 nuclei were present. Minimal numbers of neutrophils were present in the section. Based on this description, a diffuse, chronic LGC was diagnosed (Figure 1). Following the biopsy, topical dexamethasone ophthalmic ointment was recommended twice daily, ongoing.

The cat was presented to Ohio State University College of Veterinary Medicine (OSUCVM) ophthalmology specialty service for consultation 13 months after the initial biopsy diagnosis. The owners reported that there had been rapid progression of eyelid swelling OD over the previous 3–4 days. At that visit, the neuro‐ophthalmic exam found a positive menace response OU, normal direct and consensual pupillary light reflexes and dazzle reflex, and intact palpebral reflexes OU. There appeared to be normal movement of the globe OU. Intraocular pressure (IOP) values were considered normal at 20 mmHg OD and 17 mmHg OS (iCare TonoVet, iCare, Vantaa, Finland). Neither a Schirmer tear test nor a fluorescein stain test were performed. Slit lamp biomicroscopy (Kowa SL‐17; Kowa Company Ltd.) exam findings described lesions OD as a large nonpainful superior palpebral subconjunctival lid mass and smaller proliferative temporal conjunctival mass, both suspected to be lipogranulomatous based on a lobular yellow‐tan appearance. Mild conjunctival hyperemia and nictitans elevation were present OD. The visible cornea and remainder of the anterior segment appeared normal, but the details of the posterior segment were not well visualized due to the large superior eyelid mass. Findings were similar but with less marked eyelid swelling and a normal globe OS. Dexamethasone ophthalmic solution (0.1%) was prescribed three times daily OU and surgical debulking with histopathology was planned.

Four weeks later, the cat was presented for surgery. There had been a significant increase in the mass effect of the upper eyelid OD, now measuring approximately 4 cm × 4 cm with desiccation of palpebral conjunctiva with associated ulceration, crusting, and blood‐tinged discharge. The findings OS were unchanged from the prior visit. Surgical debulking of the superior eyelid mass OU included sharp and blunt dissection using Stevens tenotomy scissors to remove as much abnormal tissue as possible. The mass OD extended posteriorly along the palpebra toward the conjunctival cul de sac and was firmly adhered to the underlying eyelid tissue. No clinically normal palpebral conjunctiva was identified following mass removal OD. The bulbar conjunctiva was tacked to the underlying tissue using 6–0 Vicryl with a continuous pattern to prevent orbital fat prolapse. A lateral partial temporary tarsorrhaphy was placed using 6–0 polypropylene in a horizontal continuous pattern. Similar but less extensive debulking was performed OS. Postoperative treatment with topical oxytetracycline and polymyxin‐B sulfate ophthalmic ointment (Terramycin®) OU TID and robenacoxib (Onsior®) 1.14 mg/kg PO for 2 days were prescribed.

The debulked tissues from OU were placed in separate containers of 10% buffered formalin and submitted to the OSUCVM anatomic pathology service. The tissue was processed for histology by routine methods, and 4‐μm sections were stained with hematoxylin and eosin (H&E) stain. The superficial conjunctival epithelium OS had a regionally extensive erosion. The conjunctival substantia propria was replaced by multifocal, variably sized lipid lakes lines by fine fibrovascular stroma, numerous neutrophils, and giant lipid‐laden macrophages, consistent with LGC. Approximately 80% of the debulked tissue from OD consisted of amphophilic to eosinophilic fibrous matrices with moderately organized bundles and streams of fibroblasts. Multifocally, amphophilic to lightly basophilic myxomatous matrices accounted for approximately 20% of the debulked tissues. The only discernable conjunctival epithelium had multifocal superficial erosion overlying variably sized lipid droplets. While the superficial substantia was diffusely infiltrated by numerous neutrophils, lymphocytes, and plasma cells, the deep propria had focal lymphoid aggregate surrounding remnants of atrophied sebocytes. Focally, lipid‐laden macrophages as well as multinucleated giant cells containing up to 14 nuclei, were present in the deep propria, resembling the previously reported LGC lesions. Histopathology after the surgical debulking was consistent with the initial biopsy result from 13 months prior.

**FIGURE 2:**
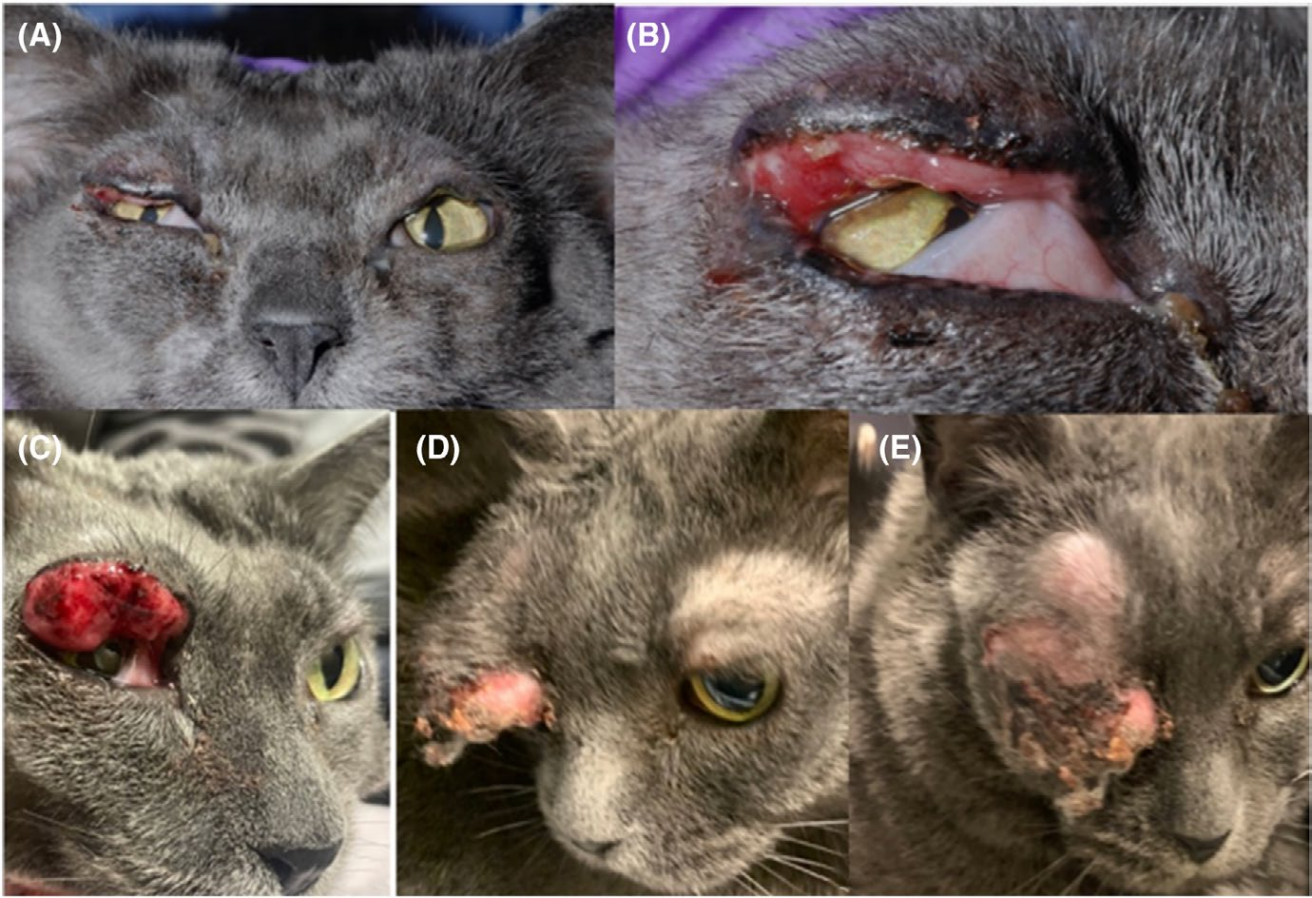
Clinical images. (A, B) Two weeks following surgical debulking OU with firm proliferative lipogranulomatous tissue along upper eyelid OU with mild contracture at the surgical site. (C) Ten days after images in (A, B), following a tapering course of oral prednisolone. (D, E) One month after images in (A, B), on presentation for exenteration OD.

At a recheck visit 2 weeks following the debulking procedure, the owners reported the cat to be pawing at the eyes and appearing to be uncomfortable. Clinical exam showed firm, proliferative lipogranulomatous tissue along upper eyelid OU with mild contracture at the surgical site (Figure 2A,B). A tapering course of oral prednisolone (1.1 mg/kg PO once daily for 14 days, followed by 0.55 mg/kg PO once daily for 7 days) was prescribed. On recheck examination 10 days later, the mass OD had grown significantly (Figure 2C). Intralesional triamcinolone (1 mg) was injected into the superior eyelid OD and it was discussed that surgical exenteration would be recommended if the lesion failed to respond. The eyelids OS were unchanged from the previous exam.

**FIGURE 3:**
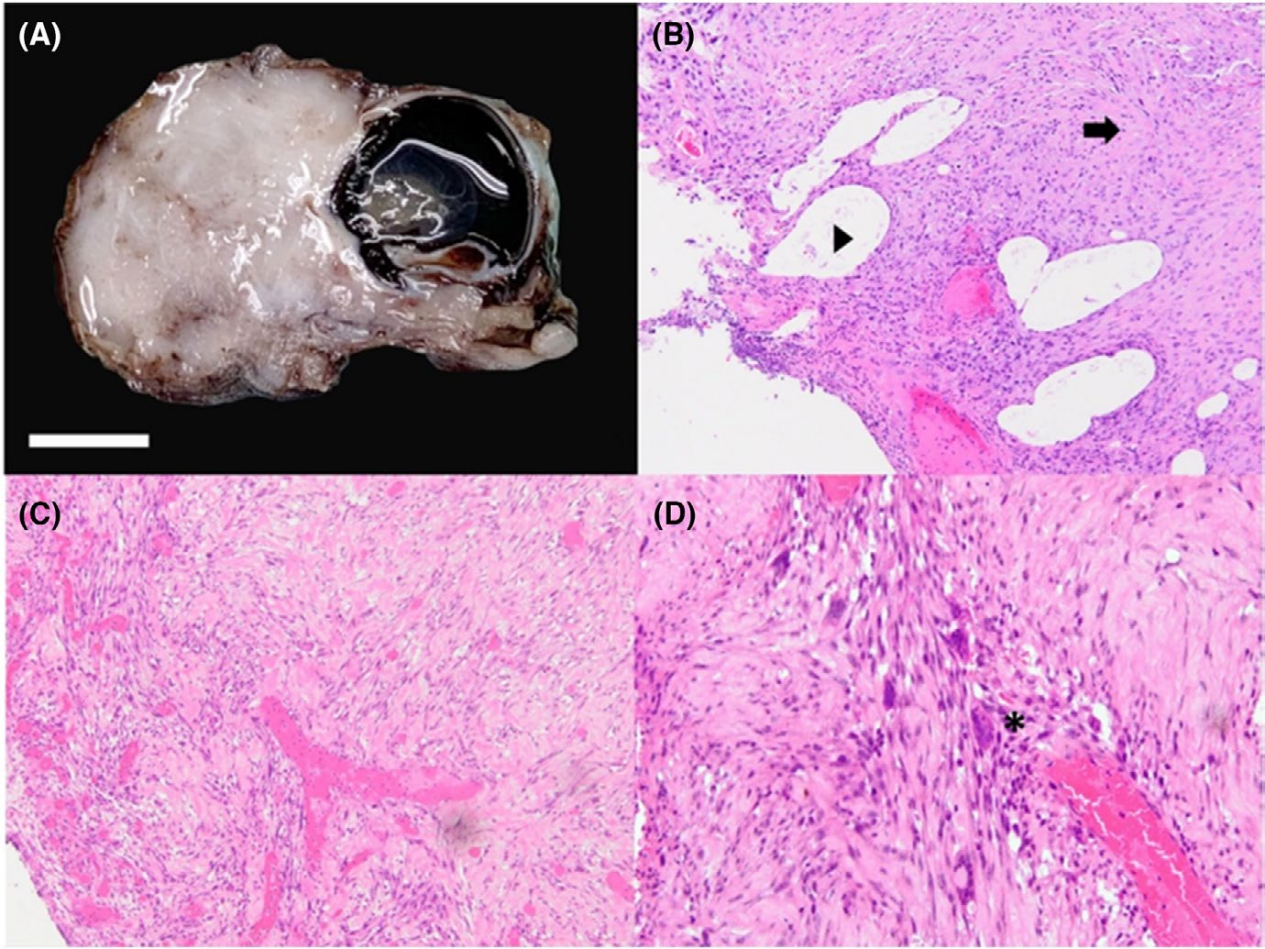
Histopathological images. (A) Gross image of a well‐demarcated, expansile fibrosarcoma arising from the superior eyelid OD. Samples taken at time of exenteration OD. (B) Tissue showing robust proliferation of fibroblasts in variably amphophilic to lightly eosinophilic fibrous stroma (arrow) with multifocal clear spaces (arrowhead) 10X. (C) Fibrosarcoma characterized by severely disorganized and pleomorphic fibroblasts embedded in dense fibrous matrix 10X. (D) Numerous multinucleated cells containing up to 14 nuclei (asterisk) 20X.

One month following surgical debulking, exenteration was performed OD (Figure 2D,E). The globe and adnexal tissues were placed in 10% buffered formalin and submitted to the OSUCVM anatomic pathology service. The globe was processed for histology by routine methods as described above. Histopathology confirmed neoplastic changes consistent with fibrosarcoma (Figure 3A–D). A gross examination of the exenterated tissue revealed severe expansion of the superior eyelid by a moderately firm pale, tan irregular mass of tissue measuring approximately 2.5 × 3 cm. On cut sections, the mass was homogeneously white and firm and covered the anterior aspect of the cornea with no evidence of invasion of the cornea. Microscopically, the mass elevated and expanded the epidermis and the dermis of the superior eyelid, causing focal erosion of the superficial eyelid epithelium. The non‐encapsulated, infiltrative and moderately cellular neoplasm was arranged in streaming bundles of mesenchymal cells supported by abundant amphophilic immature collagenous stroma, which were organizing into mature collagen fibers in some areas. The neoplastic cells had abundant eosinophilic elongated cytoplasm with indistinct cell borders with moderate anisocytosis and anisokaryosis. There were 15 mitotic figures in ten 40× (2.37 mm^2^) fields. Multifocally, there were multinucleated giant cells containing up to 15 nuclei. Toward the periphery of the neoplasm were moderate amounts of lymphoplasmacytic and neutrophilic infiltration. The orbicularis muscle bundles facing the margins of the neoplasm were multifocally hypereosinophilic, fragmented, and variably sized, attributed to focal necrosis. The remainder of the globe was essentially normal.

Oncology referral was discussed and declined. The patient was euthanized approximately 3 months following exenteration due to tumor regrowth OD. At that time, the conjunctival abnormalities of the OS had not progressed. A necropsy was not performed.

## 3 DISCUSSION

This case report is the first to describe fibrosarcoma arising within the eyelid tissue of a cat with chronic LGC. Previous reports have described LGC as being most common in light‐colored or white cats with a presumptive etiology of UV radiation inciting damage to the meibomian tissue and resulting in lipogranulomatous inflammation.^1^, ^2^ There have been five cases of neoplasia associated with LPG reported in the literature, with three of those cases being squamous cell carcinoma.^1^, ^2^ While ocular and periocular sarcomas are uncommon in cats, the presentation of this cat is not typical for either LGC or sarcoma behavior.^5^, ^6^ It is unknown if the chronic inflammation from LGC incited malignant transformation of resident mesenchymal cells or if the neoplastic changes began well prior to presentation to the ophthalmology service. In a human case report, intraepithelial sebaceous carcinoma of the conjunctiva and skin of the eyelid was reported to have slowly progressed over 10 years in conjunction with low‐grade, lipogranulomatous inflammation.^7^ Cats in particular have been described as having a propensity to developing fibrosarcoma following inflammatory events, such as at injection sites, secondary to chronic inflammatory bowel disease or following ocular trauma.^8^, ^9^, ^10^, ^11^, ^12^, ^13^, ^14^ Additionally, trauma, including surgical trauma in fracture repair, has been associated with the development of osteosarcoma in a cat.^15^ In humans, inflammation has been associated with neoplasia. Oxidative stress, inflammatory mediators, epigenetic alterations, and subsequent proliferative changes likely play a role in the development of cancer.^16^ In the current case, it is unknown if the chronic inflammation or the surgical trauma of a biopsy procedure could have triggered the malignant change.

Another uniquely feline entity, feline restrictive orbital myofibroblastic sarcoma (FROMS), is not uncommonly reported in cats.^15^, ^16^, ^17^, ^18^, ^19^ With FROMS, there is a slow progression of neoplastic changes to the spindle cell population and marked collagen deposition.^15^, ^16^ The current case differs both clinically and histopathologically from FROMS. The cat in the current case report did not have restriction of the globe movements and the reported histopathologic sample did not show the bland spindle cell population nor characteristic collagen changes along fascial planes typically seen in FROMS.^15^, ^16^ While the current case exhibited a discrete mass formation OD, FROMS tends to display diffuse thickening of the periocular tissues and may even expand into the oral cavity.

While neither the biopsy from the original visit 2 years prior to the presentation to the ophthalmology service, nor the biopsy taken during the surgical debulking procedure showed clear evidence of neoplasia, it is possible that earlier changes may have been overlooked. Biopsies from multiple sites, including eyelid margin or skin rather than just from the palpebral conjunctiva and substantia, may have provided a more complete representation of the clinical picture. Additionally, a CT scan or MRI of the area may have revealed the more aggressive nature of the disease. The owner's ultimate decision for exenteration rather than further diagnostic investigation or additional treatment was based on financial limitations. Prior to exenteration, there was clinical suspicion of malignancy based on the rapid change to the periocular tissues, so this radical surgery was considered a palliative option to alleviate discomfort in addition to an opportunity to potentially remove all diseased tissue.

This case report describes a cat with chronic, bilateral LGC that developed a unilateral fibrosarcoma within the eyelid tissue of the right eye. Fibrosarcoma should be considered a differential diagnosis in any cat with chronic LGC that develops a rapidly progressive mass in the eyelid.

## AUTHOR CONTRIBUTIONS

**T. M. Foster:** Data curation; writing – review and editing. **G. M. Newbold:** Conceptualization; data curation; supervision; writing – original draft; writing – review and editing. **E. J. Miller:** Data curation; supervision; writing – review and editing. **Y. J. Jeong:** Data curation; writing – review and editing. **C. Premanandan:** Data curation; writing – review and editing. **B. D. Husbands:** Data curation; writing – review and editing.

## CONFLICT OF INTEREST STATEMENT

The authors have no relevant conflicts of interest to declare.

## ETHICS STATEMENT

This report complies with the Guidelines for Ethical Research in Veterinary Ophthalmology (GERVO) and is exempt from approval by an ethics committee.

## Data Availability

The data that support the findings of this study are available on request from the corresponding author. The data are not publicly available due to privacy or ethical restrictions.
